# Lesion-symptom mapping reveals differential relationships between language and precise versus approximate numeracy

**DOI:** 10.1016/j.cortex.2025.10.008

**Published:** 2025-11-06

**Authors:** Erin Duricy, Corrine Durisko, Julie A. Fiez

**Affiliations:** a Learning Research and Development Center, University of Pittsburgh, Pittsburgh, PA, 15260, USA; b Center for Neuroscience, University of Pittsburgh, Pittsburgh, PA, 15260, USA; c Center for the Neural Basis of Cognition, University of Pittsburgh, Pittsburgh, PA, 15260, USA; d Department of Psychology, University of Pittsburgh, Pittsburgh, PA, 15260, USA; e Department of Communication Science and Disorders, University of Pittsburgh, Pittsburgh, PA, 15260, USA

**Keywords:** Acalculia, Aphasia, Numeracy, Lesion method, Neuropsychology

## Abstract

Numeracy, the foundation of mathematical processing, can be divided into two subcategories: approximate (quantity estimation) and precise numeracy (exact quantity). Loss of numeracy is commonly comorbid with aphasia following stroke, yet little is known about the neural basis of this relationship. We employed a support vector regression lesion-symptom mapping (SVR-LSM) analysis with N = 104 left hemisphere chronic stroke survivors to investigate the neural relationship between approximate and precise numeracy with language ability. Our results highlight key differences between how approximate and precise numeracy overlap with language processing regions. Approximate numeracy does not show a significant relationship to aphasia or language related regions, while precise numeracy shares considerable neural overlap with language areas and co-varies significantly with aphasia severity. The results support *a priori* hypotheses that the intraparietal sulcus (IPS) is crucially involved in approximation and additionally implicate regions including angular gyrus (AG), precentral gyrus, and anterior fusiform gyrus. In contrast, precise numeracy was linked to damage to the inferior frontal gyrus (IFG), AG, and anterior temporal cortex, as well as the caudate nucleus, thalamus, and posterior temporal regions. Overall, we provide evidence of strong lesion-deficit associations demonstrating distinct neural patterns between precise and approximate numeracy impairments. Crucially, we demonstrate that these subcategories have contrasting relationships with language processing.

## 1. Introduction

Numeracy, the ability to understand and manipulate quantity, is a critical skill at the foundation of mathematical processing. From estimations of relative distance and time to exact calculations in monetary purchases, cooking, and counting, numeracy underlies crucial daily life functions. As a result, impairment in numeracy ability negatively impacts employment opportunities, social and emotional outcomes, and overall quality of life (Benn et al., 2022; Bynner & Parsons, 2005; KPMG, 2008; OECD, 2012). Roughly 30 % of stroke survivors experience numeracy impairments in the form of acalculia (Zukic, Mrkonjic, Sinanovic, Vidovic, & Kojic, 2012), comparable to the estimated 20–40 % of individuals who experience language impairments, or aphasia (Engelter et al., 2006). Despite its prevalence as a comorbidity of aphasia, acalculia has been studied to a lesser degree and the neural relationship between these deficits has been explored with limited neuroanatomical specificity. Prior case series have consistently reported that global aphasia is associated with the most severe numerical deficits (Delazer, Girelli, Semenza, & Denes, 1999; Gonzalez, Rojas, Rosselli, & Ardila, 2021; Proios, Tsakpounidou, Karapanayiotides, Priftis, & Semenza, 2021; Rosselli & Ardila, 1989). Fewer studies have examined the neural relationship of such deficits and report conflicting findings. One study identified greater severity of acalculia in left hemisphere retrorolandic lesion cases (Rosselli & Ardila, 1989), while another pointed to greater numerical errors occurring as a result of left anterior lesions than posterior lesions (Proios et al., 2021). There is also some discrepancy in the degree to which acalculia and aphasia are dependent on the same brain regions, as some researchers point to a strong relationship between language and numeracy neurally (Baldo & Dronkers, 2007; Delazer & Bartha, 2001), while others highlight a dissociation between the two (Basso, Burgio, & Caporali, 2000; Fedorenko & Varley, 2016). Overall, it is important to understand the underlying neural substrates implicated in impairments of numeracy ability to better understand how they relate to the well-studied language regions of the brain that are implicated in aphasia.

### 1.1. Approximate and precise numeracy

Numeracy is often divided into two subcategories of numerical processing ability: approximate and precise numeracy (Feigenson, Dehaene, & Spelke, 2004). Approximate numeracy refers to the ability to estimate differences in magnitude without defining the exact number. This skill is thought to be innate because evidence of magnitude estimation has been found in human babies, as well as other species (Flombaum, Junge, & Hauser, 2005; Gilmore, Attridge, & Inglis, 2011; Meck & Church, 1983; Odic & Starr, 2018; Vallortigara, 2017; van Marle, Chu, Li, & Geary, 2014). Approximation does not rely on a symbolic number system and has been identified in groups of people who lack an exact counting structure within their language (Pica, Lemer, Izard, & Dehaene, 2004), suggesting an independence from language processing. In fact, the Approximate Number System (ANS) model considers approximation to be foundational to learned mathematical skills, though acuity of approximation can be enhanced with formal education (Odic & Starr, 2018; Piazza, Pica, Izard, Spelke, & Dehaene, 2013).

In contrast, precise numeracy is the ability to assign specific values to quantity. Precise numeracy emerges during development and is scaffolded by developing language as magnitudes are “mapped” onto verbal number words when learning to count (Benavides-Varela, Butterworth, et al., 2016; Carey, 2009; Gelman, Gallistel, & Gelman, 2009). Similar to language, precise numeracy is a symbolic system. Symbolic representations of quantity (e.g., Arabic digits) correspond to exact quantities, and these can be used in a learned syntax to complete mathematical operations (Delazer & Bartha, 2001; Seron, 2001). Thus, measures of precise numeracy include counting, transcoding (converting a value from one form to another, such as the verbal word “one” to the digit “1”), and calculation. Precise numeracy is distinct from underlying approximation, such that children may struggle with accessing magnitude from symbolic representations, but not in comparing magnitudes themselves (Rousselle & Noël, 2007). Therefore, approximate and precise numeracy follow distinct developmental trajectories and likely differ in their neural and behavioral relationships to language ability.

### 1.2. Neuroanatomical theory

The Triple Code Model proposed by Dehaene (1992) remains the prevailing theory of numerical processing. It posits that three representational codes interact bidirectionally to form numerical processing in the brain (Dehaene, 1992; Dehaene & Cohen, 1995). The *visual Arabic number form* localized to the bilateral inferior ventral occipitotemporal area represents numbers visually through numerical symbols. The *verbal auditory word frame*, aligning with the left perisylvian area, represents the word form of number, underlying counting and arithmetic fact retrieval as numbers are mapped onto verbal representations. Finally, the *analog magnitude representation* code is localized to the bilateral inferior parietal lobe encompassing the intraparietal sulcus (IPS) and represents pre-verbal approximate reasoning and estimation important for magnitude comparison, estimation, or subitizing. While the Triple Code Model seeks to represent numerical processing globally, approximate and precise numeracy are implied parts of the whole. Based on this model, impairments in approximate numeracy should localize to the IPS, while precise numeracy impairments may arise from a range of possible neural substrates — from the foundational magnitude representation of the IPS to the broader, language-centric perisylvian area.

### 1.3. Empirical evidence

Theoretical advancements in the numeracy literature, including the Triple Code Model, emerged primarily from neuropsychological approaches. Single-case studies and case series have provided foundational evidence for numerical processing, but are both limited in quantity and crude in nature — typically identifying broad regions of the brain (e.g., left parietal lobe) or lacking generalizability (Boller & Grafman, 1983; Dehaene & Cohen, 1995, 1997; Dehaene, Piazza, Pinel, & Cohen, 2003; Grafman, Passafiume, Faglioni, & Boller, 1982). More recent efforts to expand lesion-deficit understandings of numeracy have been made, but remain highly limited. Recently, we published a meta-analysis comprising a systematic search of parietal single case studies to extract generalizable numeracy data from single cases (Duricy, Durisko, & Fiez, 2025). We found that, relative to lesions in other areas of the parietal lobe, cases with lesions to left IPS exhibited a greater frequency of approximation impairment (Duricy et al., 2025). To date, only one known voxel-based lesion-symptom mapping (VLSM) analysis has tackled the question of a numeracy and language relationship (Baldo & Dronkers, 2007), while limited others focus on specific sub-abilities of numeracy like transcoding (Benavides-Varela, Passarini, et al., 2016; Haupt, Gillebert, & Demeyere, 2017). Using a narrower scope of behavioral performance in their VLSM, Baldo and Dronkers (2007) found that damage to left IFG was associated with both language comprehension and arithmetic, and damage to left AG was associated with impaired arithmetic performance specifically. Overall, there is a need for larger scale lesion studies with greater neuroanatomical specificity to parse out the neural correlates of acalculia.

The majority of recent advances in the localization of numeracy are derived from functional imaging studies. The IPS is considered to be crucial for foundational elements of magnitude representation, based on the Triple Code and Approximation Number System models (Dehaene & Cohen, 1995; Dehaene et al., 2003; Odic & Starr, 2018). Evidence for bilateral IPS activation during non-symbolic magnitude tasks is robust (Ansari & Dhital, 2006; Bloechle et al., 2018; Holloway, Price, & Ansari, 2010; Lyons, Ansari, & Beilock, 2015; Venkatraman, Ansari, & Chee, 2005), supporting its role in approximate numeracy performance. Left-lateralized IPS activation, however, has been linked to symbolic magnitude comparison using both numerals (Bugden, Price, McLean, & Ansari, 2012; Matejko, Hutchison, & Ansari, 2019; Vogel et al., 2017) and number word stimuli (Lussier & Cantlon, 2017). Additionally, there is evidence of IPS contribution to precise numeracy skills. Bilateral IPS activation has been shown during calculation tasks (Bugden et al., 2012, 2019; Chochon, Cohen, van de Moortele, & Dehaene, 1999; Molko et al., 2003; Suárez-Pellicioni, Soylu, & Booth, 2021). Further, a reduction of gray matter volume in left IPS was found to be linked to reduced transcoding skills in children (Lubin et al., 2013), though two lesion-symptom mapping studies did not find a correlation between IPS and transcoding impairment in an adult sample (Benavides-Varela, Passarini, et al., 2016; Haupt et al., 2017). Overall, these results suggest that IPS may be involved in precise numeracy processing as well as approximate numeracy processing.

The angular gyrus (AG) has been strongly linked to arithmetic fact retrieval and exact calculation (Andin, Fransson, Rönnberg, & Rudner, 2015; Dehaene et al., 1999, 2003; Delazer et al., 2003; Faye et al., 2019; Grabner, Ansari, et al., 2009; Grabner, Ischebeck, et al., 2009; Skagerlund et al., 2019). Bilateral AG activation has been linked to exact calculation relative to approximate calculation (Dehaene et al., 1999). Further, AG was highlighted as a parietal counterpart to the perisylvian area's verbal auditory word frame in a later variation of the Triple Code Model because of its involvement in the verbal manipulation of number (Dehaene et al., 2003). This is in line with support for the left AG's role in language processing, which primarily points to AG's involvement in semantic processing (Kim et al., 2022; Price, 2000; Van Ettinger-Veenstra, McAllister, Lundberg, Karlsson, & Engström, 2016), as well as cross-modal skills of higher cognition employed in both language and numerical processing (Seghier, 2013).

Additionally, the left inferior frontal gyrus (IFG) has strong connections to language processing and speech production but also shows activation during precise numerical processing. In language literature, there is particular support for activation of the *pars triangularlis* and *pars opercularis* subdivisions that make up Broca's Area during speech production (Dapretto & Bookheimer, 1999; Ishkhanyan et al., 2020; Keller, Crow, Foundas, Amunts, & Roberts, 2009; Klaus & Hartwigsen, 2019; Medaglia et al., 2021). In numeracy literature, multiple studies link left IFG activation to number naming, arithmetic performance, and developmental math achievement (Chochon et al., 1999; Dehaene et al., 1999, 2004; Delazer et al., 2003; Faye et al., 2019; Skagerlund et al., 2019; Wilkey & Price, 2019). Andin et al. (2015) assessed left IFG in the context of both arithmetic and language phonology, finding that left IFG *pars triangularis* demonstrated activation during both phonological tasks and multiplication and subtraction operations.

### 1.4. Overview of study

There is currently a large gap in our understanding of acalculia and how it relates to aphasia. The present study begins to fill in this gap through assessment of lesion—deficit relationships of numeracy by employing a more refined neuroanatomical approach with a considerable sample size. We employ a support vector regression lesion-symptom mapping (SVR-LSM) analysis to examine whole-brain patterns of impairment in precise versus approximate numeracy, aiming to better understand their neural correlates and their relationships to language processing. Based on theoretical foundations and empirical evidence, we hypothesize that approximate numeracy performance will be impacted by damage to the left IPS, in particular, and will not co-vary with aphasia severity. In contrast, we hypothesize that precise numeracy performance will be impaired following damage to any or all of our expected regions — left IPS, AG, or IFG — and that precise numeracy will co-vary with aphasia severity. Finally, we expect that additional regions may emerge as significant contributors of numerical processing, given the ability of SVR-LSM to examine patterns of correlations across all voxels.

## 2. Materials and methods

### 2.1. Participants

A total of 104 left hemisphere (LH) stroke survivors participated in this study (see Table 1 for demographics). For SVR-LSM analyses, a sample size of 100–120 has been shown to be acceptable for prediction accuracy and reproducibility (Sperber, Wiesen, & Karnath, 2019, pp. 1065–9471). All participants were recruited from the Western Pennsylvania Research Registry, a database of stroke survivors interested in research participation (Balason, Durisko, & Fiez, 2018; Fiez & Behrmann, 2024). Participants were recruited based on the following criteria: 1) a single, unilateral left hemisphere lesion acquired at least 6 months previously, 2) right-handed, native English speakers with no history of a learning disability or developmental neurological disorder, 3) no history of other major neurological disorders, or a medical problem that would preclude study participation, and 4) able to provide informed consent and understand simple written or spoken instructions. Participants were not excluded on the basis of lesion size or lesion location within the left hemisphere. All participants voluntarily consented to participate in the study following the University of Pittsburgh Institutional Review Board approved procedure.

### 2.2. Behavioral testing methods

The behavioral tasks utilized in this study were administered as part of a broad language, math, and cognition battery. The full battery was administered across 1–2 testing sessions, lasting approximately 3–5 h with breaks. Due to the timeline in which behavioral testing was completed, the battery was administered fully in-person for 44 participants, but adopted to a virtual administration protocol via a HIPAA-compliant version of Zoom (Zoom Video Communications Inc. Security Guide, 2016) for the remaining 60 participants following the onset of the COVID-19 pandemic. Based on our previous work assessing the reliability of virtual testing relative to in-person testing with stroke survivors (Duricy, Durisko, Dickey, & Fiez, 2023; Durisko, McCue, Doyle, Dickey, & Fiez, 2016), as well as systematic reviews of remote neuropsychological test administration (Batastini, Paprzycki, Jones, & MacLean, 2021; Brearly et al., 2017; Brown & Zakzanis, 2023; Dekhtyar, Braun, Billot, Foo, & Kiran, 2020; Despoti et al., 2024), we are confident that the modality of testing has not significantly impacted the quality of data acquired.

In the present study, data were drawn from a subset of the full testing battery to focus on tasks involving numerical processing. It should be noted that none of the numerical tasks required verbal responses so as to reduce the impact of aphasia on task performance. The numerical tasks included in this study for analysis are described briefly below. The battery also included the full Western Aphasia Battery (WAB) (Kertesz & Raven, 2007) as a standardized assessment of aphasia, from which aphasia quotient scores were derived.

#### 2.2.1. WAB auditory word recognition (numerical items only)

Participants are presented with a page with six numbers, ranging from 1 to 4 digits and written as Arabic numerals. The administrator dictates one number, and the participant must point to the respective number in digit form (Kertesz & Raven, 2007). All six items of this task are visible at once and the participant must select each item as it is read aloud. This task is a subtest of the standardized WAB. It was scored following WAB guidelines, with 1 point given for each correct item, 0 points for each incorrect item, and 0 points if the participant points to more than one choice unless clearly self-correcting.

#### 2.2.2. WAB supplemental number writing and dictated numbers

Participants are asked to write the numbers 0–20 on a piece of paper in digit form. Participants are then asked to write six specific numbers that range from 1 to 4 digits, dictated by the administrator (Kertesz & Raven, 2007). These items were scored following WAB guidelines. For writing 0–20, .5 points were given for each correct number within the 0–20 range, with a maximum score of 10. Numbers were also scored correctly even if they were written out of order. The maximum score value allows for one incorrect number, according to the standard scoring rules. For dictated number writing, 1 point was given for each correct dictated number written and 0 points were given for each incorrect number.

#### 2.2.3. WAB supplemental calculation

Participants are shown 12 simple (1–2 digit) math problems with four multiple choice answer options. They are asked to indicate, either orally or through pointing, what is the correct answer to the equation. They are not allowed to solve the problems with pen and paper. There are three items per operation: addition, subtraction, multiplication, and division (Kertesz & Raven, 2007). These items were scored following the WAB guidelines, with 2 points given for each correct answer and 0 points given for each incorrect answer or no response.

#### 2.2.4. Comprehensive aphasia test (CAT) calculation

Participants are shown seven math problems, ranging from 1 to 3 digits, with five multiple choice answer options. Items include three addition, three subtraction, and one multiplication equation. Participants are asked to indicate the correct answer to each equation (Swinburn, Porter, & Howard, 2005). They are not allowed to solve the problems with pen and paper. This task was scored following CAT guidelines, with 1 point given for each correct answer and 0 points given for each incorrect answer or no response.

#### 2.2.5. Computer-based dot comparison task

In this non-symbolic task, participants are presented with two dot clouds simultaneously for 750 msec on a screen, and then must press the Left or Right arrow key to indicate which of the two quantities had a greater magnitude (Halberda, Mazzocco, & Feigenson, 2008; Libertus, Feigenson, & Halberda, 2013; Lyons & Beilock, 2009; Mejias, Grégoire, & Noël, 2012; Mejias, Mussolin, Rousselle, Grégoire, & Noël, 2012). Quantities vary by fixed ratios, dot size, and congruity, with a total of 96 trials. This task is run in PsychoPy v2020.2.3 (Peirce et al., 2019). Scores are calculated based on performance accuracy, with 1 point for a correct response and 0 points for an incorrect response.

#### 2.2.6. Behavioral statistical analysis

To directly investigate approximate and precise numeracy performance, each task was first scored for accuracy, then the raw scores within each task were *z*-transformed. Then, a composite Precise Numeracy measure was created for each participant, by averaging together their scores from the WAB auditory number word recognition, WAB number writing and dictated numbers, WAB calculation, and CAT calculation tasks. The dot comparison z-score was used to provide a pure assessment of Approximate Numeracy as a non-symbolic, magnitude-driven skill. To assess language ability, an aphasia quotient (AQ) was derived from the WAB assessment for all participants, where a higher score (≥93.8) indicates normal language ability and decreasing scores correspond to increased aphasia severity.

### 2.3. Imaging methods

We collected research-grade structural magnetic resonance images (MRIs) for a total of 80 participants. For an initial cohort of participants (n = 32), they were acquired with a Siemens 3T Allegra scanner using a single channel head coil. This imaging protocol included a high-resolution T1 MPRAGE (TR 1540 msec, TE 3.04, 192 slices, 1 mm isotropic voxels, and 256 mm FoV) and a T2 FLAIR image (TR 6200 msec, TE 353, 160 slices, 1 mm isotropic voxels, and 220 mm FoV). For the remaining participants (n = 48), they were collected on a Siemens 3T Prisma scanner with a 60 cm bore using a 32-channel head coil (Carnegie Mellon University - CMU-Pitt BRIDGE Center). The protocol included a high-resolution T1 MPRAGE (TR 2300 msec, TE 2.03 msec, 208 slices, 1 mm isotropic voxels, and 256 mm FoV), T2 FLAIR (TR 6000 msec, TE 388, 208 slices, 1 mm isotropic voxels, and 256 mm FoV), and T2 images (TR 3000 msec, TE 294 msec, 176 slices, 1 mm isotropic voxels, and 256 mm FoV).

For the remaining participants (n = 24), research-grade images were not collected due to delays from the COVID-19 pandemic and contraindications. For this group of participants, clinical MR and computed tomography (CT) scans were retrieved from the participants’ medical records, with consent provided as part of their enrollment into WPPR. Within the set of images available for each case, the clinical image selected for lesion segmentation was collected at the most chronic timepoint obtainable (>3 months if available), contained minimal artifacts, and had the greatest spatial resolution based on reduced slice thickness.

### 2.4. Lesion segmentation and processing

Lesions were segmented on the research-grade T1-weighted brain volumes when available, and otherwise using the best available MR or CT clinical-grade brain volumes. All lesions were manually traced using ITK-SNAP (Yushkevich et al., 2006). All segmentations were checked by a neurologist (G. W.) and colleague (F.L.), who were blinded to the behavioral results and patient information. Structural images were normalized to a standard 1 mm^3^ reference brain in (ICBM_Avg152) using enantiomorphic registration (Nachev, Coulthard, Jäger, Kennard, & Husain, 2008; Rorden, Bonilha, Fridriksson, Bender, & Karnath, 2012) in MATLAB R2022b (TheMathWorksInc., 2022). This approach uses the segmented lesion mask to temporarily mask the lesion in native space during registration, which improves the quality of lesioned tissue in the normalized image (Nachev et al., 2008).

### 2.5. Support vector regression lesion-symptom mapping analysis

We employed multivariate support vector regression lesion-symptom mapping (SVR-LSM) to examine lesion—behavior relationships from a brain-wide perspective. This analysis was chosen because multivariate LSM considers patterns of voxelwise correlations rather than the individual voxel-by-voxel approach of univariate methodologies, so multiple regions can be considered simultaneously for a more nuanced, accurate assessment of lesion—behavior relationships. We utilized the SVR-LSM toolbox with graphical user interface in MATLAB R2022b for all lesion-symptom mapping analyses (DeMarco & Turkeltaub, 2018). In order to account for tissue damage beyond the visible lesion and to mitigate the hard cutoff points of binarized lesion data resulting from lesion segmentation, we applied a Gaussian smoothing kernel of 4 mm full width half maximum to all normalized lesion masks before their inclusion in the analysis (Ivanova, Herron, Dronkers, & Baldo, 2021). We used the default SVR-LSM hyperparameters recommended for optimal performance (C = 30, γ = 5) (DeMarco & Turkeltaub, 2018; Zhang, Kimberg, Coslett, Schwartz, & Wang, 2014). A voxel threshold of 5 participants was selected to best encompass areas of the brain that are less frequently impacted by stroke but remain theoretically relevant to numerical processing.

We ran two LSM models (Model A, Model B) for each of our numeracy measures (Precise, Approximate). In each case, Model A did not include aphasia severity, while Model B included aphasia severity as an expected predictor of numeracy performance in order to parse out clusters influenced by a significant relationship to language processing. Demographic covariates of age and years of education were included in all models. As recommended for all SVR-LSM analyses, we also included lesion volume as a covariate in all models (DeMarco & Turkeltaub, 2018; Ivanova et al., 2021; Zhang et al., 2014). The set of covariates was used as regressors for both behavioral and lesion data in all models, as recommended (DeMarco & Turkeltaub, 2018).

Model A: Numeracy Performance = β_o_ + β_1_Voxel Overlap + β_2-_Years of Education + β_3_Age + β_4_ Lesion Volume

Model B: Numeracy Performance = β_o_ + β_1_Voxel Overlap + β_2-_Years of Education + β_3_Age + β_4_ Lesion Volume + β_5_Aphasia Quotient

We ran one-tailed SVR-LSM analyses for both approximate and precise numeracy, where negative SVR-β values correspond to impaired performance. To identify significant voxel and clusterwise relationships, we tested 5000 permutations of the resulting SVR-β values. Voxelwise significance was thresholded to *p* < .005, and clusterwise was thresholded to *p* < .05.

## 3. Results

**Fig. 1 —:**
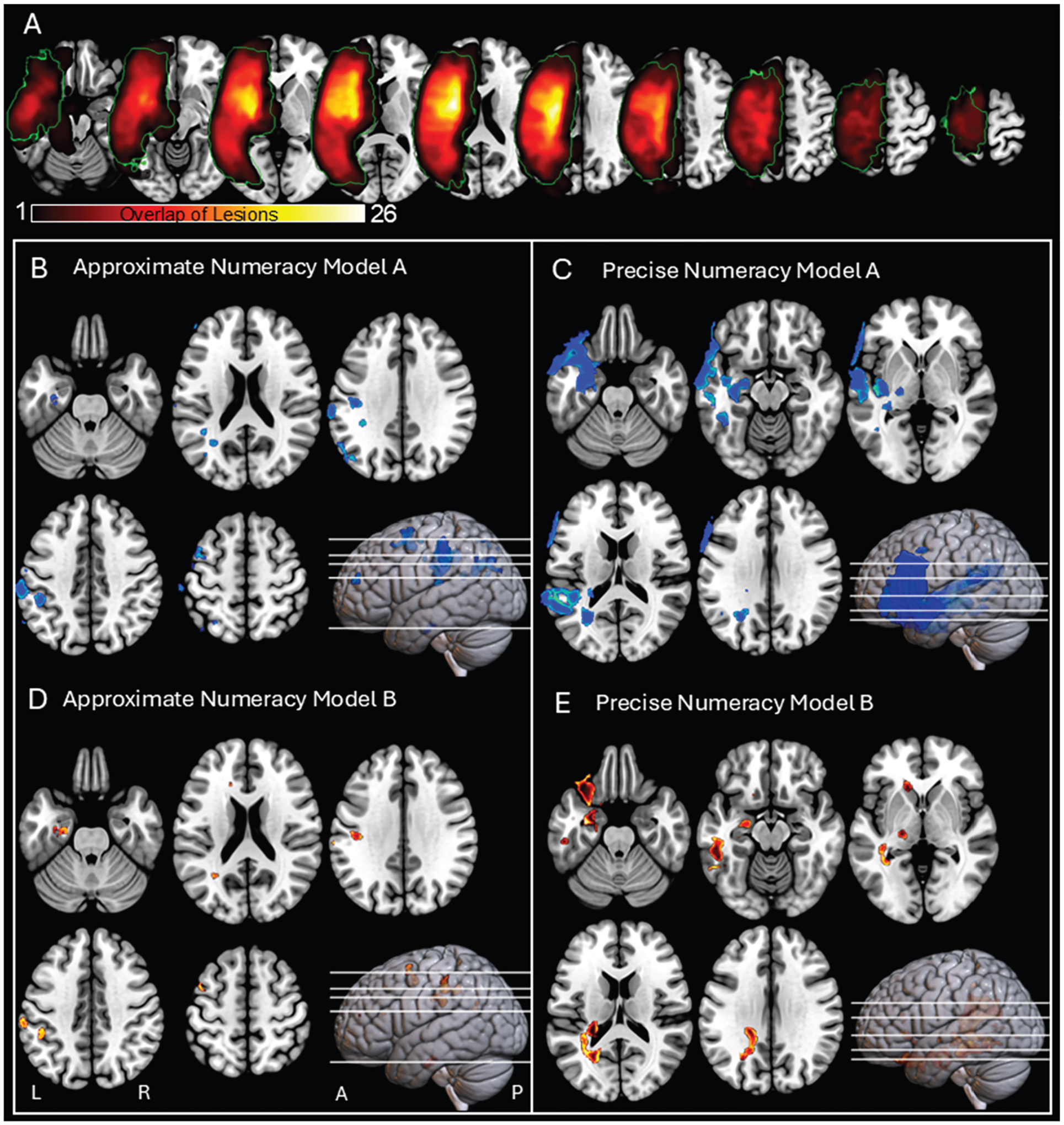
Results of the support vector regression lesion-symptom mapping (SVR-LSM) analyses. Total overlap of lesions included in all models, with the significant voxel threshold outlined in green (A). Model A (blue) and Model B (orange) significant voxelwise results for the Approximate (B,D) and Precise (C,E) measures, with slices highlighting key voxel groups.

The results of the SVR-LSM analysis revealed key differences in the brain regions implicated in impaired approximate and precise numeracy performance. Table 2 contains the significant voxelwise results and whether each cluster survived clusterwise correction. Fig. 1 visualizes the significant voxelwise results of each model. The total volume of voxels assessed in all SVR-LSM analyses is indicated in panel 1A, demonstrating broad left hemisphere coverage with the largest degree of overlap in the perisylvian area. It should be noted that one participant was excluded from the analysis based on a lesion location that did not overlap within the threshold boundary, so the final analysis included a total N = 103 participants.

Overall, approximate and precise numeracy deficits largely localize to disparate regions. Approximate numeracy impairments were associated with significant voxels (*p* < .005) localized primarily to the parietal lobe in both Model A (Fig. 1B) and Model B (Fig. 1D), while precise numeracy impairments largely localized to the temporal lobe in both Model A (Fig. 1C) and Model B (Fig. 1E). None of the Approximation results survived clusterwise correction (*p* ≥ .22), while Precise Model A resulted in one significant cluster (*p* = .0004) that was eliminated in Precise Model B.

Interestingly, we found significant voxel overlap with key theoretical and empirically supported regions, as well as distinct regions beyond those traditionally discussed in the context of numerical processing. Crucially, approximate numeracy deficits aligned with the anterior IPS, a key magnitude processing region identified across studies. Additionally, across both models, approximate numeracy impairments were associated with damage to the inferior parietal lobule including the supramarginal gyrus and AG, the precentral gyrus, portions of parieto-occipital white matter, and anterior fusiform gyrus (with some extension to parahippocampal gyrus). The inclusion of aphasia severity as a covariate did not affect the pattern of significant voxels for approximate numeracy, with consistent voxel localizations between models. The centers of mass (CM) of the largest clusters in Approximate Model A (−64, −29, 40) and B (−63, −30, 44) were localized to comparable Montreal Neurological Institute (MNI) coordinates (Table 2). The consistency of localization results across models indicates that approximate numeracy impairment results from brain damage that is independent of language ability.

Precise numeracy impairments localized to both the IFG and AG, key regions previously linked to both language and numerical skills. However, in contrast to the findings for approximate numeracy, these associations were only observed in Model A, in which aphasia severity was not included as a covariate. Once language ability was included for consideration of variance in the data, the large cluster encompassing IFG and the anterior temporal lobe, which originally survived both voxelwise (*p* < .005) and clusterwise correction (*p* = .0004), was now eliminated at both levels. For comparison, the center of mass from the significant cluster in Precise Model A (−50, −7, −5) did not align with the coordinates of the largest cluster result in Precise Model B (−26, −45, 19) (Table 2). Interestingly, a different pattern of significant voxels emerged in Precise Model B, which revealed significant voxels in the posterior temporal region and caudate nucleus. This result indicates that precise numeracy is not wholly contingent on language ability, rather it relies on regions both intertwined and distinct from language performance. Overall, precise numeracy impairments were associated with lesions in the inferior and middle temporal gyri, AG, IFG, temporal pole, parahippocampal gyrus and hippocampus, the thalamus, head of the caudate nucleus, and cerebral white matter underlying the temporo-parieto-occipital junction. These findings demonstrate a clear relationship between language and precise numeracy by revealing a covaried relationship at known language-related areas of the brain, as well as highlighting the complexity of precise processing through localizations to subcortical regions and temporal areas independent of aphasia severity.

**Fig. 2 —:**
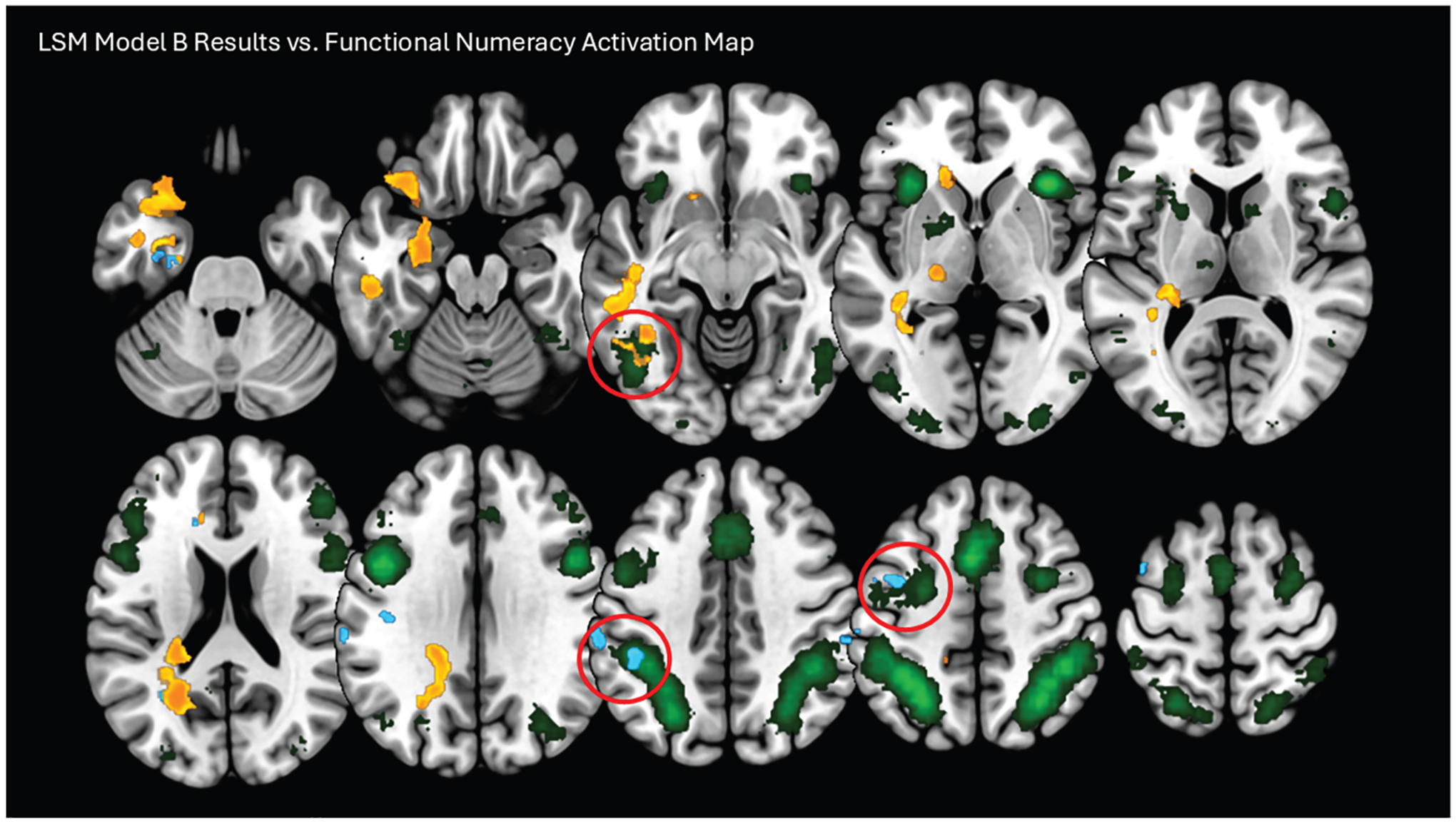
Model B voxelwise results overlaid with functional numeracy meta-analytic activation map. Functional map derived from Neurosynth Topic 018 v5-topics-100 uniformity map. Areas of voxel overlap circled in red. Approximate numeracy = cyan, Precise numeracy = orange, Functional map = green.

To contextualize our lesion findings within prior functional numeracy research, we overlaid the results of Approximate and Precise Model Bs with a meta-analytic activation map of numerical processing (Fig. 2). Model B was selected for this comparison as a more direct representation of numerical processing specifically, with all covariates accounted for. The activation map was extracted from Neurosynth, a meta-analytic open-source functional neuroimaging platform (Yarkoni, Poldrack, Nichols, Van Essen, & Wager, 2011). The activation map used (Topic 018 v5-topics-100) included results from 260 studies and was labeled with top-loading terms like “numerical”, “magnitude”, and “counting”. As shown in Fig. 2, our lesion results converge with multiple activation clusters identified across prior functional neuroimaging studies of numerical processing. Approximate numeracy deficits overlapped with activation of IPS and precentral gyrus, and impaired precise numeracy overlapped with inferior and middle temporal gyri activation. By converging our lesion-deficit approach with prior functional imaging findings, we provide strong support for the functional relevance of these three regions in numerical processing performance. Further, our results emphasize the anatomically distinct nature of approximate versus precise skills, demonstrating a clear distinction in the elements of numeracy underlying several of the activation clusters identified across prior functional neuroimaging literature.

## 4. Discussion

**Fig. 3 —:**
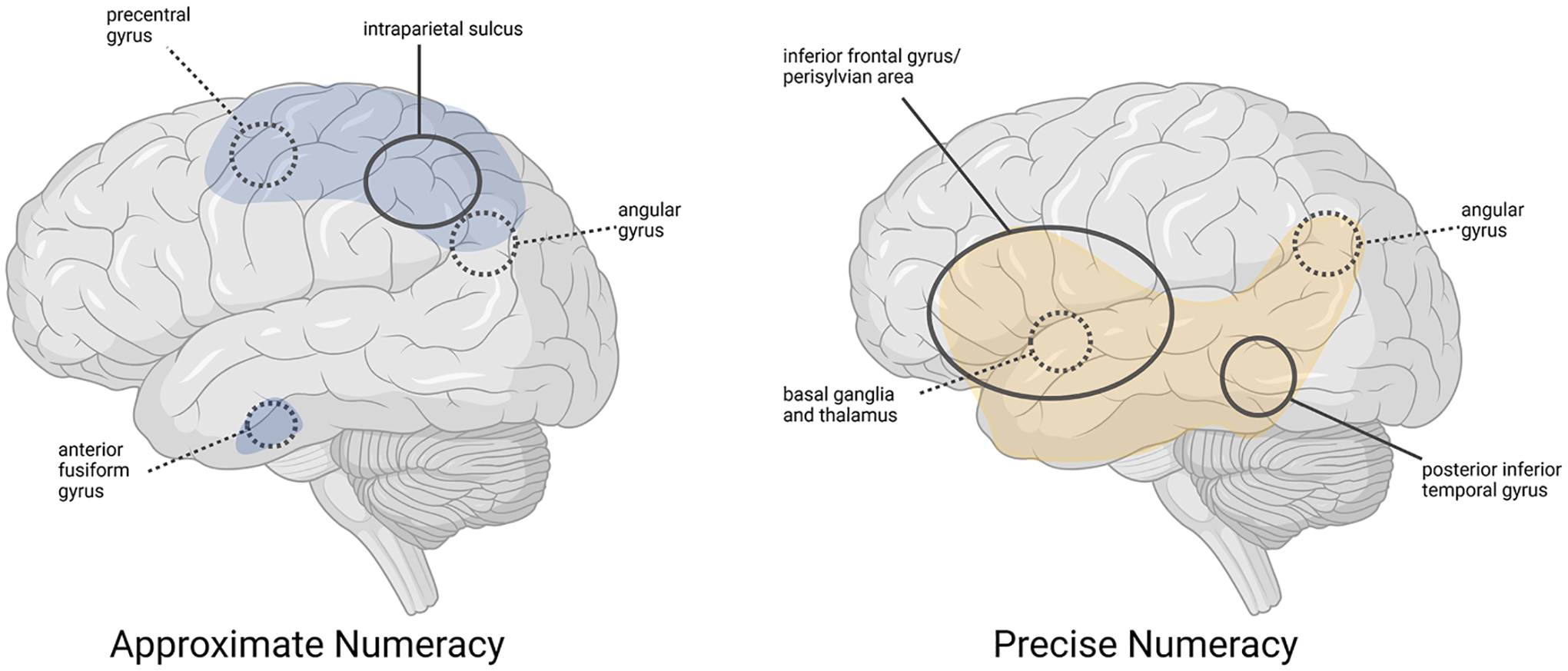
Schematic of anatomical distribution of precise and approximate numeracy processing. Precise numeracy area shaded yellow; approximate numeracy area shaded blue. Solid lines indicate regions supported by the Triple Code Model (Dehaene & Cohen, 1995), while dashed lines indicate areas we propose as additional sites of importance. This figure was created in BioRender (Duricy (2025) https://BioRender.com/lecv1yr).

Impairments in numeracy and language are frequently comorbid following stroke, but little is known about the extent to which different forms of numeracy are encompassed in this relationship or how this relationship maps to common sites of neural damage. To address this gap in knowledge, we employed the first known lesion-symptom mapping assessment of precise versus approximate numeracy, and we adjusted our SVR-LSM models to elucidate the impact of aphasia severity on each. We find key differences in the neural correlates underlying impaired performance of precise and approximate numeracy. Crucially, we also identify distinct relationships between each type of numeracy and language ability. As predicted, approximate numeracy is associated with damage to the left IPS. Additionally, we found evidence for associations with surrounding inferior parietal lobe regions including the AG, as well as the precentral gyrus and anterior fusiform gyrus. Approximate numeracy deficits are independent of language impairment and are not associated with damage to language regions. Contrastingly, precise numeracy is linked primarily, but not exclusively, to regions involved in language processing. Precise numeracy deficits are highly correlated with aphasia severity and our results highlight the partially overlapping nature of the two processes. Precise numeracy is associated with damage to language-dependent areas including IFG, AG, and anterior temporal cortex, as well as independent regions including inferior and middle temporal gyri and several subcortical regions. To synthesize our anatomical results in relation to the Triple Code Model's foundational localization of numerical processing, we provide a diagram highlighting our key findings (Fig. 3).

### 4.1. Neural substrates for approximate numeracy

The results for approximate numeracy were comparable regardless of whether aphasia severity was included as a factor, with significant voxel groups localized to locations that were both visually consistent and shared similar centers of mass between models. This leads us to conclude that approximate numeracy and language processing are not directly linked and rely on largely distinct brain regions.

Overall, we find convergent support for the role of the left IPS in non-symbolic approximation ability as outlined in the Triple Code Model and Approximate Number System (Dehaene & Cohen, 1995; Odic & Starr, 2018) and supported by numerous functional imaging studies (Ansari & Dhital, 2006; Escobar-Magariño, Turel, & He, 2022; Faye et al., 2019). The IPS has long been linked to non-symbolic magnitude estimation as a pre-verbal *analog magnitude representation* code in both the original and parietal-focused Triple Code Model proposals (Dehaene & Cohen, 1995; Dehaene et al., 2003). These models were drawn from select single case studies encompassing lesion cases, split brain patients, and hemispherectomies (Dehaene & Cohen, 1995), suggesting that the IPS is necessary for approximation ability. In a recent meta-analysis of parietal lesion single case studies, we found that approximation impairment localized similarly to the left posterior IPS and inferior parietal lobe (Duricy et al., 2025). However, we also noted that only 50 % of the identified left IPS lesion cases demonstrated impaired approximation performance, suggesting that damage to left IPS alone may not be sufficient for complete loss of approximate numeracy ability. This may be due to involvement of the right hemisphere in numerical processing. The bilateral IPS is linked to magnitude representation (Dehaene & Cohen, 1995) and activation of both left and right IPS have been associated with approximation performance in functional imaging studies (Ansari & Dhital, 2006; Bloechle et al., 2018; Chochon et al., 1999; Escobar-Magariño et al., 2022; Kucian et al., 2006; Piazza, Izard, Pinel, Le Bihan, & Dehaene, 2004). Our study focuses only on unilateral left hemisphere lesions, which means that compensation from the contralateral hemisphere could play a role in partial preservation of approximation abilities. Additionally, other regions beyond the IPS may contribute to approximate numerical processing and thus may account for this discrepancy in deficit profiles. The present findings shed light on several regions beyond the IPS that could fill this role within the left hemisphere.

Within the parietal lobe, our results also implicated left AG in approximate numeracy. While we expected AG involvement in precise numeracy, we did not expect an association with approximation. Previously, we found that the left angular gyrus was associated with impaired precise numeracy skills like ordinality/cardinality and transcoding, but not approximation, in a group of parietal lesion cases (Duricy et al., 2025). It is possible that AG has an indirect effect on approximate numeracy performance, rather than playing a foundational role. The left AG is considered a hub, linked to numerous white matter tracts and involved in a variety of higher cognitive processes (Seghier, 2013). The AG is typically considered to be a language center, but AG has also been implicated in spatial cognition that could play a role in approximate numeracy (Sack, 2009). Based on the fact that the left AG voxels were significant both before and after aphasia severity was accounted for in the models, we expect that these results are not dependent on the language role of AG. Instead, spatial cognition processing in the AG may impact performance on the dot comparison task, which involves visuo-spatial assessment of two dot clouds with varying sizes and spread of dots. Additionally, there are proposed functional distinctions between anterior and posterior AG (also referred to as PGa and PGp), which are both structurally distinct in neuronal architecture (Niu & Palomero-Gallagher, 2023) and have demonstrated different roles in semantic cognition processing (Seghier, Fagan, & Price, 2010). Our approximate numeracy results localize to the posterior AG, while our precise numeracy Model A cluster localizes to the anterior AG, which aligns with an anterior-posterior phonological to semantic gradient that has been proposed in the inferior parietal lobule (Humphreys & Lambon Ralph, 2015; Humphreys & Tibon, 2023). An alternative possibility is that this finding may be due to the anatomical proximity of the AG and IPS in the parietal lobe, where lesions that impact the IPS are more likely to partially extend to AG and vice versa. In fact, a recent electrocorticography study demonstrated the high likelihood that significant AG results in group studies of mathematical cognition may actually be due to anatomical boundary blurring from close proximity to the IPS (Pinheiro-Chagas, Chen, Sabetfakhri, Perry, & Parvizi, 2023). Finally, we cannot rule out the possibility that expertise in learned precise skills improves innate magnitude estimation skills, thereby negatively influencing approximation performance when brain regions primarily involved in precise processing are damaged. It is possible that AG, situated within the parietal lobe and acting as a hub of connections for a variety of cognitive processes, may contribute to this effect.

Outside of the parietal cortex, we found precentral gyrus involvement, which notably overlapped with meta-analytic results from functional neuroimaging studies of numerical processing. While not addressed in the prevailing theories of numeracy, the precentral gyrus has been previously linked to functional numerical processing, including activation during non-symbolic number comparison (Wilkey, Barone, Mazzocco, Vogel, & Price, 2017), number relational processing (Zhang et al., 2019), and functional connectivity to IPS during arithmetic (Skagerlund et al., 2019). Given the convergence of our precentral gyrus cluster with meta-analytic results from prior numeracy studies, we suggest that the left precentral gyrus may be an additional component of the neural substrate for approximation processing.

Additionally, we found approximate numeracy deficits localized to the anterior fusiform gyrus. The mid-fusiform gyrus is often considered in the context of face and object recognition and reading (Balsamo, Xu, & Gaillard, 2006; Ghuman et al., 2014; McCandliss, Cohen, & Dehaene, 2003). The area we identified is located more anteriorly, within a portion of the temporal lobe sometimes referred to as the basal temporal language area (BTLA). The BTLA is linked to language performance, primarily comprehension and object naming (Krauss et al., 1996), but one prior study found impaired performance on written calculation during electrical stimulation of the region (Lüders et al., 1991). Crucially, multiple patients could not remember the problem they attempted or which digits were included following stimulation, suggesting that this area may contribute to broader identification and memory of numerical values (Lüders et al., 1991). Additionally, prior imaging studies have linked the fusiform gyrus to calculation and non-symbolic comparison (Arsalidou & Taylor, 2011; Ashkenazi, Black, Abrams, Hoeft, & Menon, 2013). There is also limited but compelling evidence that the entire length of the fusiform gyrus may be directly connected to IPS through an association fiber tract (Jitsuishi & Yamaguchi, 2020), further supporting the possibility of a left anterior fusiform gyrus contribution to magnitude approximation. Overall, we provide strong lesion-deficit support for additional regions beyond IPS that may be vital to approximate numeracy processing, but have been largely overlooked in broader literature. Further investigation of these regions is needed to better understand their roles in approximate numeracy specifically.

### 4.2. Neural substrates for precise numeracy

Precise numeracy impairment showed a distinctly different neural pattern than approximation and, crucially, a pattern strongly tied to language impairment. Where approximation models revealed largely consistent patterns of significant voxels, precise Models A and B show drastic differences dependent on aphasia severity as a covariate. Based on the Triple Code Model, precise numeracy should align with regions in the left perisylvian area to constitute the *verbal auditory word frame* that underlies verbal components of number processing (Dehaene, 1992; Dehaene & Cohen, 1995). Further, prior empirical findings using functional imaging suggest the involvement of language-related regions in precise numerical processing (Delazer et al., 2003; Grabner, Ansari, et al., 2009; Kim et al., 2022; Klaus & Hartwigsen, 2019; Seghier, 2013; Wilkey & Price, 2019). We provide converging support for this prediction, finding significant associations of precise numeracy with left IFG and superior temporal cortex extending to the anterior AG. Importantly, these results were entwined with language ability, such that these regions only constituted a significant cluster when aphasia severity was *not included* as a variable in Model A. Therefore, damage to these regions is linked to precise numeracy impairments, but in conjunction with impairments in language ability. This pattern reflects the results of Baldo and Dronkers (2007), to our knowledge the only other LSM analysis to approach a question of numeracy versus language processing. In that study, arithmetic and language comprehension were compared at the neural level, and arithmetic alone was associated with damage to supramarginal and angular gyri of the inferior parietal lobe, while language comprehension was broadly associated with middle and superior temporal gyri. Crucially, the two processes overlapped in the inferior frontal gyrus and areas within middle and superior temporal gyri (Baldo & Dronkers, 2007), mapping closely to the significant cluster identified in Model A.

It is important to discuss, however, how our findings relate to evidence of an opposing perspective in which language is independent of other cognitive processes like arithmetic. Fedorenko and colleagues have proposed a linguistic network that is distinct from other higher order cognitive processes, and report several instances in which arithmetic performance did not significantly correlate to perisylvian language regions (Fedorenko, Ivanova, & Regev, 2024; Fedorenko, Piantadosi, & Gibson, 2024; Fedorenko & Varley, 2016). They provide support from a functional imaging region-of-interest analysis which reported deactivation of language regions during easy and hard math localizer trials (adding three consecutive addends to a two-digit number to compute a final sum) relative to rest, as well as a whole brain analysis that showed frontoparietal activation in a hard > easy arithmetic contrast (Fedorenko, Behr, & Kanwisher, 2011). Further support includes a positron emission tomography study that found deactivation of perisylvian regions during arithmetic computation relative to rest (Zago et al., 2001), a functional study of algebraic syntax that showed anatomical separation from linguistic syntax (Monti, Parsons, & Osherson, 2012), and a study reporting three left hemisphere lesion cases that did not exhibit deficits in computational ability (Varley, Klessinger, Romanowski, & Siegal, 2005). What these studies have in common, and what may be the source of discrepant findings between this framework and our reported results, is a focus on computational ability versus our broader focus on precise numeracy, with less computationally demanding calculation. Computational ability refers to the ability to solve calculation problems algorithmically by following learned rules and procedures, and this is typically associated with more complex calculation tasks (Fuchs et al., 2008). Though it relies on precise quantity knowledge to understand and compute exact quantities, computational ability has been shown to be linked to nonverbal processing impairments, such as spatial cognition, procedural knowledge, and working memory (Fuchs et al., 2008; Geary, 1993; Rourke & Finlayson, 1978). These nonverbal cognitive abilities are more often linked with parietal regions (Basso et al., 2000; Sack, 2009), the multiple demand network (Duncan, 2010; Fedorenko, Duncan, & Kanwisher, 2013), or even activation of the right hemisphere (Salillas, Benavides-Varela, & Semenza, 2023; Semenza & Benavides-Varela, 2018). In contrast, our precise numeracy measure includes arithmetic fact calculation tasks as well as number transcoding tasks. We intentionally include multiple types of tasks to assess performance on tasks requiring broad precise number knowledge. Performance impairment on both calculation and transcoding has been correlated to aphasia in a prior case series of 50 left hemisphere patients (Delazer & Bartha, 2001). Even in a case series reporting a dissociation between calculation and aphasia, there is a clear relationship between language ability and number transcoding ability (Basso et al., 2000). Therefore, we suggest that our results may be disparate from the framework of Fedorenko and colleagues largely because of differences in the tasks selected to represent numeracy ability. Our focus on precise numeracy includes less computationally demanding calculation and a wider range of precise numerical tasks, including transcoding, which may influence the localizations we find here. Future studies would benefit from a closer examination of how numeracy task impacts neuroanatomical localizations of numerical processing, but at present we find evidence for the partial overlap of language processing and precise numeracy processing in the brain, encompassing the left IFG and perisylvian area.

We also acknowledge that there have been reports of right hemisphere damage associated with impairment in precise numerical tasks, including single digit calculation (Benavides-Varela et al., 2014; Semenza, Benavides-Varela, & Salillas, 2025) and number transcoding (Benavides-Varela, Passarini, et al., 2016; Haupt et al., 2017; Semenza et al., 2025). There is some question as to the role of the right hemisphere in these traditionally language-related tasks, given that language is widely considered to be left hemisphere dominant (Vigneau et al., 2006). The prevailing interpretation is that the right hemisphere provides non-linguistic contributions to support precise numerical processing, such as processing the spatial arrangement of an operand or relating a precise number or operation to a mental number line. A future direction for this work, and for the field of numeracy as a whole, is to better understand the extent of right hemispheric contribution to precise numeracy.

Outside of the left perisylvian area, we found evidence for additional left hemisphere regions with contributions to precise numeracy that were effectively “unmasked” once the model accounted for language variance. Precise Model B results localized voxel clusters in the posterior inferior temporal gyrus, white matter underlying temporal cortex, the temporo-parieto-occipital (TPO) junction, and the thalamus and caudate nucleus. Notably, the posterior inferior temporal gyrus cluster overlapped with a meta-analytic statistical map from prior functional studies, converging to support a site of numerical processing activation that is specific to precise numeracy and independent of both approximation and language.

Prior research has proposed ventral temporal cortex involvement in precise numerical processing. One study using intracranial electroencephalography with epileptic patients found that posterior inferior temporal cortex activation occurred during arithmetic tasks, particularly bursting at the onset of the operation and thought to be associated with problem difficulty (Pinheiro-Chagas, Daitch, Parvizi, & Dehaene, 2018). Aligned with the Triple Code Model's *visual Arabic number form* code, there is also evidence of a “visual number form area,” localized to the posterior inferior temporal cortex, which is activated during the presentation of numerical digits and number words specifically (Abboud, Maidenbaum, Dehaene, & Amedi, 2015; Baek, Daitch, Pinheiro-Chagas, & Parvizi, 2018; Conrad, Pollack, Yeo, & Price, 2023; Dehaene & Cohen, 1995; Pollack & Price, 2019). Previously, this area has been localized to coordinates very closely aligning with the coordinates we observe. We identify groups of voxels with centers of mass at −52, −32, −13 (cluster 3, Precise Model B) and −43, −54, −10 (cluster 4, Precise Model B), the latter of which extends laterally as far as −59, −55, −15. These coordinates are highly similar to the localization of a left number form area in a bilateral investigation (average coordinates = −54, −55, −13) (Grotheer, Herrmann, & Kovács, 2016) and a localized right hemisphere counterpart in another (peak right hemisphere coordinates = −53, −44, −12) (Abboud et al., 2015). Limited evidence also supports the involvement of occipital-temporal regions, including the TPO junction. A recent functional connectivity study examining subtraction and multiplication skills across development identified the temporo-parieto-occipital junction as a region of interest from predictive models of multiplication (Li et al., 2024). Overall, our results support the involvement of the ventral temporal area in precise numeracy and, in line with the Triple Code Model's separation of the *visual number form* from the *verbal auditory word frame*, we find that its role is less dependent on language ability.

Additionally, we find language-independent subcortical areas that emerge in Model B. The basal ganglia has been linked to arithmetic fact retrieval and calculation procedural knowledge in limited functional neuroimaging studies (Delazer et al., 2004; Saban, Pinheiro-Chagas, Borra, & Ivry, 2024). Further, Dehaene and colleagues have previously proposed the basal ganglia and thalamus as a subcortical hub for precise processing of arithmetic facts (Dehaene & Cohen, 1997), supported by single case studies (Corbett, McCusker, & Davidson, 1986; Dehaene & Cohen, 1997; Hittmair-Delazer, Semenza, & Denes, 1994; Whitaker, Habiger, & Ivers, 1985). Our results support these findings across a much larger sample of lesion data and highlight the need for further investigation of the role of basal ganglia and thalamus in precise numeracy performance. Our results converge with evidence across methodologies to provide lesion-deficit support of precise numeracy impairments at these non-traditional numeracy regions. Further investigation of precise numeracy subskills at the whole-brain level would expand our understanding of the specific role of such regions.

### 4.3. Limitations

There are several considerations for contextualizing the current findings. First, SVR-LSM has limitations to its ability to identify significant results at a clusterwise level when voxels are widely distributed across the brain or are grouped in multiple relatively small areas, as is the case with our results (Rorden, Fridriksson, & Karnath, 2009). Though voxelwise results still provide important information about lesion—behavior relationships, it is important to consider the possibility of false positives at the level of voxelwise results, given the limited clusters that survived correction (DeMarco & Turkeltaub, 2018; Mirman et al., 2018). Additionally, we must consider the low threshold of voxelwise overlap (n = 5) that was utilized for this sample. We opted for a lower threshold than is sometimes seen in more conservative studies using n = 10, or even 10 % of a given sample, but we chose this value to encompass most of the left hemisphere, especially superior regions that are less frequently impacted by stroke. A more stringent threshold would likely reveal more robust clusterwise results in terms of statistical power, but these findings would be anatomically limited and exclude key areas of theoretical relevance.

Additionally, it is important to consider the impact of vasculature and lesion location on the interpretation of results. Because the anatomical distribution of stroke sites is non-random due to common brain vasculature and the blood flow of major arteries, damage to a given brain region often occurs in conjunction with damage to anatomically proximal regions. This can lead to a ‘topographical bias’ in lesion-symptom mapping studies, where significant voxels may represent either causal sites of behavioral impairment or anatomically adjacent sites that are only correlated with damage to a given voxel, but not the behavior itself (Sperber, 2020). Given this consideration, our results may highlight a degree of extraneous voxels that are not functionally related to precise or approximate numerical processing. However, we focus on several key regions that are scaffolded by support from prior functional imaging studies linking these regions to numerical processing. Importantly, we provide convergent evidence that at least three significant voxel groups — left IPS, precentral gyrus, and posterior inferior and middle temporal gyri — directly overlap with voxels identified across a meta-analysis of functional imaging studies of numeracy performance. This lends strong support for a direct functional role of these brain regions in precise and approximate numeracy.

The distributed pattern of significant SVR-LSM voxels suggest that multiple regions contribute to a larger network, where behavioral impairment becomes apparent when one or more nodes of a related network are damaged. There is increasing support for the role of functional brain networks in numerical processing. Recent studies of math competence in children have consistently linked both symbolic arithmetic and non-symbolic magnitude performance to activity within the frontoparietal, or “multiple demand” network (Kuhl, Sobotta, Consortium, & Skeide, 2021; Ng et al., 2024; Ren & Libertus, 2023; Stillesjö et al., 2021). This network is composed of regions highlighted in the current study, IPS and the precentral gyrus found to be linked to approximate deficits in our SVR-LSM analysis, and other regions including anterior cingulate cortex, pre-supplementary motor area, IFG *pars opercularis*, anterior insula, and frontal operculum (Assem, Glasser, Van Essen, & Duncan, 2020; Duncan, 2010, 2013). Contrastingly, the language network is composed of fronto-temporal regions that strongly align with areas identified in our precise numeracy models, specifically IFG and middle temporal gyri extending from the anterior-most point of the temporal poles to posterior temporal lobe (Fedorenko, Ivanova, & Regev, 2024). Next steps should include further investigation at the network level to examine whether precise and approximate numeracy may be utilizing largely disparate *networks* for processing beyond regional differences. Additionally, expanding the whole-brain perspective to more nuanced assessment of subskills within precise and approximate numeracy may elucidate how these abilities could emerge from one or more functional brain networks.

Finally, it must be acknowledged that the behavioral tasks included in this study were limited in terms of stimulus format. Precise numerical tasks involved only symbolic number formats (e.g., Arabic numerals or written word forms), while the approximate numerical task involved only non-symbolic stimuli (e.g., dot clouds). We used a non-symbolic stimulus task for the measure of approximate numeracy in order to assess pure magnitude comparison performance, rather than combining this data with symbolic numerical estimation tasks. This was chosen because there is some theoretical debate in magnitude estimation literature disputing differences between symbolic and non-symbolic numerical stimulus processing and the level of precise ability required for understanding symbolic numbers (Goffin & Ansari, 2019; Sokolowski et al., 2017, 2021; Zebian & Ansari, 2012), so we opted to assess approximation only through a testing format that avoided this confound. However, this decision means that we are unable to rule out the possibility that the neuroanatomical results reported here are better explained by differences between symbolic and non-symbolic stimulus processing. Future studies would benefit from investigating the transcoding of numerical information across stimulus formats to assess the relationship between task stimuli and numerical processing ability.

### 4.4. Considerations for clinical relevance

Our findings emphasize the clinical importance of acalculia interventions following stroke. The relationship between numeracy and language should be taken into consideration when assessing individuals with brain damage in both research and clinical contexts. Studies that focus on aphasia should consider the precise numerical skills of their sample, particularly in cases of significant behavioral impairment. While aphasia is assessed quickly and thoroughly following brain damage, clinical measures of precise numeracy are minimal at best, or not tested at all. Given the strong relationship between precise numeracy and language, interventions for aphasia could easily incorporate more effective interventions for improving impaired calculation, transcoding, and counting skills. This appears to be relevant regardless of the site of lesioned tissue and could significantly improve the precise numerical processing abilities of countless individuals who suffer from aphasia with undiagnosed acalculia.

### 4.5. Conclusions

In summary, we find that precise and approximate numeracy performance impairments exhibit different patterns of correlation to neural sites. Damage to key neural substrates implicated in numeracy literature — left IPS, AG, and IFG — do impact numeracy performance with differing profiles for each type of numeracy. However, comorbid behavioral impairments and damage to other regions, including prefrontal gyrus, anterior fusiform gyrus, posterior inferior temporal gyrus, and the basal ganglia and thalamus, help to better explain deficits in numerical processing. Notably, we find contrasting relationships between the two types of numeracy and language. Precise numeracy performance and aphasia severity are strongly linked following stroke, while approximate numeracy is largely independent of language. Ultimately, we provide evidence of strong lesion-deficit associations demonstrating distinct patterns of precise and approximate numeracy impairments. Crucially, we demonstrate that these subcategories have contrasting relationships with language processing regions of the brain.

## Supplementary Material

1

Supplementary data to this article can be found online at https://doi.org/10.1016/j.cortex.2025.10.008.

## Figures and Tables

**Table 1 —:** Participant Demographics.

	Age (years)	Sex	Race/Ethnicity	Education (years)	WAB Aphasia Quotient	LH total lesion volume	Time Since Stroke (months)
Mean	61.59	59 M, 45 F	95 W, 6 B, 1 NA, 2 H/L	15.07	93.02	4.45 %	67.05
SD	12.12			2.71	12.18	8.15 %	83.71

WAВ =Western Aphasia Battery, LH =left hemisphere, Races include White (W), Black or African American (B), Native American or Alaskan Native (NA), and Hispanic or Latino (H/L).

**Table 2 —:** Clusterwise results listed with descending number of voxels. Clusters reported with cutoff of N = 10 or more voxels.

Model	Cluster	P-value	N Voxels	CM x	CM y	CM z
Approximate Model A	1	.22	2720	−64	−29	40
	2	.45	933	−43	3	55
	3	.48	810	−44	−40	39
	4	.49	785	−52	−66	32
	5	.60	459	−44	−21	33
	6	.73	213	−30	−58	22
	7	.77	152	−33	−17	−27
	8	.77	149	−40	−48	22
	9	.82	83	−66	−25	16
	10	.83	78	−47	52	19
	11	.85	63	−40	−70	23
	12	.89	31	−25	−72	50
	13	.89	29	−44	−66	54
	14	.90	26	−50	−8	54
	15	.90	22	−61	−12	38
	16	.91	18	−30	−61	55
Approximate Model B	1	.60	419	−63	−30	44
	2	.61	391	−41	0	52
	3	.64	332	−29	−15	−27
	4	.65	309	−44	−21	33
	5	.66	285	−45	−40	40
	6	.77	108	−65	−28	29
	7	.78	95	−30	−57	22
	8	.86	26	−16	28	18
	9	.87	19	−55	−59	28
Precise Model A	1	<.001*	47,085	−50	−7	−5
	2	.23	2487	−28	−60	23
	3	.52	586	−44	−44	−15
	4	.67	220	−21	−18	−2
	5	.75	114	−55	9	43
	6	.78	79	−40	−58	2
	7	.82	46	−24	−36	25
	8	.84	33	−55	−50	−4
Precise Model B	1	.10	6078	−26	−45	19
	2	.15	4334	−30	9	−24
	3	.23	2651	−52	−32	−13
	4	.53	552	−43	−54	−10
	5	.62	297	−15	24	−1
	6	.69	167	−44	−5	−28
	7	.70	163	−20	−21	−1
	8	.80	49	−17	17	−8
	9	.81	42	−16	−39	47
	10	.83	28	−13	30	19
	11	.83	27	−15	17	−17
	12	.85	15	−25	−81	−3
	13	.86	13	−59	−6	−6
	14	.86	11	−36	−59	9
	15	.86	10	−24	−66	6

P-values >.05 do not survive clusterwise correction, significant clusterwise results denoted with an asterisk.

CM =Center of mass, Montreal Neurological Institute (MNI) coordinates.

## Data Availability

DATA: Some raw and processed data supporting this research are publicly available, while some are subject to restrictions: https://openneuro.org/datasets/ds006533. CODE: All analysis code supporting this research is publicly available: https://github.com/rordenlab/spmScripts/, https://github.com/neurolabusc/clinical, https://github.com/atdemarco/svrlsmgui. MATERIALS: No study materials supporting this research are publicly available. DESIGN: This article reports, for all studies, how the author (s) determined all sample sizes, all data exclusions, all data inclusion and exclusion criteria, and whether inclusion and exclusion criteria were established prior to data analysis. PRE-REGISTRATION: No part of the study procedures was pre-registered in a time-stamped, institutional registry prior to the research being conducted. No part of the analysis plans was pre-registered in a time-stamped, institutional registry prior to the research being conducted. For full details, see the *Scientific Transparency Report* in the supplementary data to the online version of this article.
