# Supplementary material for: Lesion-symptom mapping reveals differential relationships between language and precise versus approximate numeracy
Source: Cortex. Author manuscript; Available in PMC 2026 Jun 22. (PMC13285964; doi:10.1016/j.cortex.2025.10.008)
Supplement: 1 [file NIHMS2185840-supplement-1.docx]

**Scientific transparency statement**

DATA: Some raw and processed data supporting this research are publicly available, while some are subject to restrictions: <https://openneuro.org/datasets/ds006533>

CODE: All analysis code supporting this research is publicly available: <https://github.com/rordenlab/spmScripts/>, <https://github.com/neurolabusc/clinical>, <https://github.com/atdemarco/svrlsmgui>

MATERIALS: No study materials supporting this research are publicly available.

DESIGN: This article reports, for all studies, how the author(s) determined all sample sizes, all data exclusions, all data inclusion and exclusion criteria, and whether inclusion and exclusion criteria were established prior to data analysis.

PRE-REGISTRATION: No part of the study procedures was pre-registered in a time-stamped, institutional registry prior to the research being conducted. No part of the analysis plans was pre-registered in a time-stamped, institutional registry prior to the research being conducted.

For full details, see the *Scientific Transparency Report* in the supplementary data to the online version of this article.
